# Weight-Related and Personal Risk Factors of Carpal Tunnel Syndrome in the Northern Finland Birth Cohort 1966

**DOI:** 10.3390/jcm11061510

**Published:** 2022-03-10

**Authors:** Kaisa Lampainen, Rahman Shiri, Juha Auvinen, Jaro Karppinen, Jorma Ryhänen, Sina Hulkkonen

**Affiliations:** 1 Department of Hand Surgery, Helsinki University Hospital and University of Helsinki, 00014 Helsinki, Finland; jorma.ryhanen@icloud.com (J.R.); sina.hulkkonen@helsinki.fi (S.H.); 2 Finnish Institute of Occupational Health, 00032 Helsinki, Finland; rahman.shiri@ttl.fi; 3 Medical Research Center Oulu, University of Oulu and Oulu University Hospital, 90014 Oulu, Finland; juha.auvinen@oulu.fi (J.A.); jaro.karppinen@oulu.fi (J.K.); 4 Center for Life Course Health Research, University of Oulu, 90014 Oulu, Finland; 5 Rehabilitation Services of South Karelia Social and Health Care District, 53130 Lappeenranta, Finland

**Keywords:** carpal tunnel syndrome, body mass index, waist circumference, waist-to-hip ratio, obesity, median nerve

## Abstract

Background: Excess body mass is a risk factor for carpal tunnel syndrome (CTS), but the mechanisms of this are unclear. This study aimed to evaluate the association between CTS and personal risk factors of body mass index (BMI), waist circumference and waist-to-hip ratio (WHR). Methods: The study sample consisted of the Northern Finland Birth Cohort 1966 (*n* = 9246). At the age of 31 in 1997 and at the age of 46 in 2012, the participants underwent a clinical examination. Cohort A consisted of complete cases with a follow-up from 1997 to 2012 (*n* = 4701), and Cohort B was followed up from 2012 to 2018 (*n* = 4548). The data on diagnosed CTS were provided by the Care Register for Health Care until the end of 2018. Results: After an adjustment for confounding factors, BMI was associated with CTS among women (hazard ratio (HR) 1.47, 95% Cl 0.98–2.20 for overweight women and HR 2.22, 95% Cl 1.29–3.83 for obese women) and among both sexes combined (HR 1.35 95% Cl 0.96–1.90 for overweight and HR 1.98 95% Cl 1.22–3.22 for obese participants). Neither waist circumference nor WHR was associated with CTS. Conclusions: BMI is an independent risk factor for CTS and is more relevant for estimating the increased risk of CTS due to excess body mass than waist circumference or WHR.

## 1. Introduction

Carpal tunnel syndrome (CTS) is the most common entrapment neuropathy of the upper extremities and carpal tunnel release is the most common surgical procedure for the upper extremities [1,2,3]. CTS causes work disability and a great economic burden [4,5]. Based on previous studies, the incidence rate of CTS per 100,000 person-years varies between 88 and 105 among men and 193 and 232 among women, and these rates increase until middle age among both genders [6,7,8].

Well-known risk factors for CTS are age, female gender, overweight, diabetes mellitus and thyroid disease [7,9,10,11,12,13,14,15,16]. Arthritis, pregnancy and hand trauma are also potential risk factors for CTS [15,16,17]. The role of smoking as a risk factor for CTS is unclear [18].

Both overweight and obesity are also risk factors, but the mechanism of this is unclear [19]. Only a few case-control and cross-sectional studies have investigated the relationship between waist circumference and CTS [12,20,21,22] and found an association between the two. In their case-control study, Mondelli and colleagues (2014) showed that a high waist-to-hip (WHR) ratio (>0.95 for men and >0.85 for women) is an independent risk factor for CTS. They found that obese participants (BMI ≥ 30) were at an increased risk of CTS despite their WHR, whereas overweight participants (BMI 25–29.9) were only at risk if their WHR was high [20]. A previous longitudinal study found no association between waist circumference or WHR and carpal tunnel release after controlling for confounding factors [23].

This large birth cohort study aimed to evaluate the association between CTS and personal risk factors, including BMI, waist circumference and WHR.

## 2. Materials and Methods

### 2.1. Study Population

**Figure 1:**
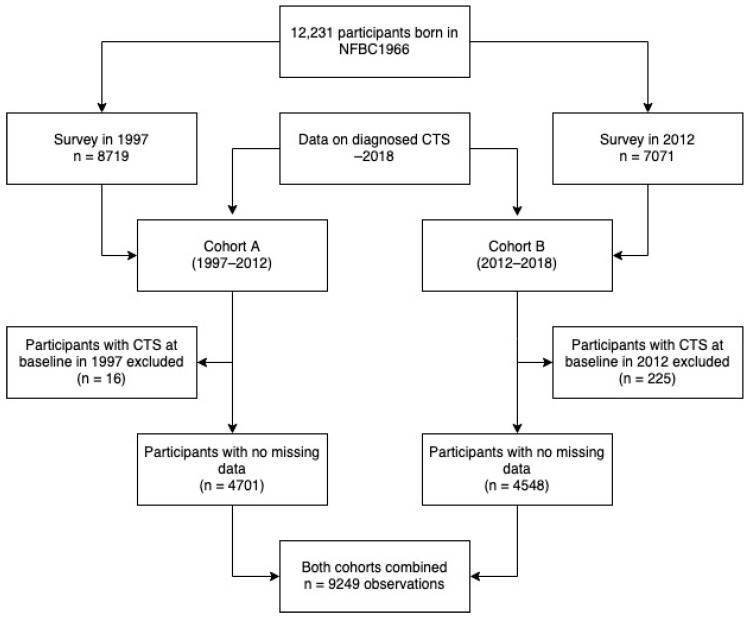
Flowchart of NFBC1966 study population.

The study population consisted of the Northern Finland Birth Cohort 1966 (NFBC1966), which originally consisted of 12,231 participants with an expected date of birth in 1966, born in the Oulu and Lapland provinces [24]. These cohort participants have been studied at several time points throughout their lives. We used data collected in 1997, when they were aged 31 (baseline population, cohort A) and in 2012, when they were 46 (follow-up population, Cohort B) (Figure 1) [25]. When handling the data, we replaced each participant’s personal identification number with a study identification code. The study was approved by the Northern Ostrobothnia Hospital District Ethical Committee 94/2011 (12 December 2011), and followed the principles of the Declaration of Helsinki.

### 2.2. Cohort A (1997–2012)

In 1997, at the age of 31, a total of 8719 participants gave their informed consent to voluntarily participate in the study, underwent a clinical examination, and answered several questionnaires. Of this study population, 16 participants were already diagnosed with CTS and were excluded from the analysis. Of the 8703 participants, 4701 with no missing data were included in the study.

### 2.3. Cohort B (2012–2018)

The second follow-up study was conducted in 2012 when the cohort was 46 years old. In total, 7071 participants gave their written consent to voluntarily participate in the study, underwent a clinical examination, and answered several questionnaires. Of these, 225 participants were diagnosed with CTS before the second follow-up study and were excluded. Finally, 4548 participants were complete cases and were included in the study.

### 2.4. Data Collection 1997 and 2012

The participants attended the clinical examination and answered several questionnaires. We measured their weight and height, their waist and hip circumference and calculated their body mass index (BMI) and WHR. BMI, waist circumference and WHR were divided into three categories according to WHO: normal (18.5–24.9 kg/m^2^), overweight (25.0–29.9 kg/m^2^) and obese (>30 kg/m^2^); low risk (<94 cm for men and <80 cm for women), intermediate risk (94–102 cm for men and 80–88 cm for women), and high risk (>102 cm for men and >88 cm for women); low risk (≤0.95 for men and ≤0.80 for women), intermediate risk (0.96–1.0 for men and 0.81–0.85 for women), and high risk (>1.0 for men and ≥0.86 for women), respectively.

Socio-economic status was defined according to Statistics Finland’s Classification of Socio-economic Groups 1989 [26]. This classification divides people into nine categories: farmers, entrepreneurs, upper and lower clerical workers, manual workers, students, pensioners, the unemployed and the unknown. The socio-economic status variable was formed by the following groups: (1) upper clerical workers, (2) lower clerical workers, (3) entrepreneurs, (4) farmers and manual workers (combined), and (5) students, pensioners, and unemployed (combined). If the status was coded as unknown, it was handled as missing data. Information on regular smoking, diabetes, rheumatoid arthritis and thyroid diseases was collected. Cohort A included complete cases and was followed up from 1997 to 2012 (*n* = 4701), and Cohort B from 2012 to 2018 (*n*= 4548), forming a total study sample of *n* = 9249.

### 2.5. Data on Diagnosed Carpal Tunnel Syndrome

The data on diagnosed CTS were provided by the Care Register for Health Care, which is a national register covering public and private hospital data in Finland. It identifies over 95% of hospital discharges and 80–99% of common diagnoses [27]. It contains information on patients’ demographic characteristics, diagnoses, surgical procedures and admission and discharge dates. The diagnoses are coded according to the International Classification of Diagnoses (ICD). According to the eighth revision of ICD 1981–1986, CTS was coded as 357.2; in line with the ninth revision of ICD 1987–1995, as 354.0; and according to the tenth revision in 1996–2016, CTS was coded as G56.0. The diagnoses were obtained from hospital data covering inpatient and outpatient data in specialist care. In specialist care in Finland, the diagnosis of CTS is based on clinical findings and positive electroneuromyography (ENMG) findings.

### 2.6. Statistical Analyses

The Cox proportional hazards regression model was used to study the association between baseline characteristics and CTS, controlled for panel data. First, we ran sex-specific age-adjusted models or age- and sex-adjusted models for both sexes combined. An association was considered statistically significant if the 95% confidence interval (CI) of the hazard ratio (HR) did not include 1. In these cases, the variables associated with CTS were added to the full models. BMI, waist circumference and WHR were added to the models one at a time. Next, both BMI and waist circumference were added simultaneously to the models. Finally, a multiplicative interaction between BMI (continuous variable) and the other baseline factors was tested.

## 3. Results

The mean follow-up time was 14.69 (SD 1.66) years for Cohort A, 4.48 (SD 0.41) years for Cohort B and 9.67 (SD 5.25) years for both cohorts combined. A total of 290 participants (3.1%) were diagnosed with CTS during follow-up. The incidence of CTS was higher among women than among men, as, during the follow-up, 4.0% of women and 2.2% of men were diagnosed with CTS. We also found that 51.7% of the study population had increased BMI. In the univariable analysis of both genders combined, overweight and obesity measured by BMI, increased waist circumference, and increased WHR were also associated with CTS (Table 1). The results of the sex-specific analyses for women were similar to those for both genders combined. Among men, obesity and increased waist circumference were associated with CTS (Table 2).

In the multivariable analysis, BMI was associated with hospitalization for CTS among women and among both sexes combined (Table 3). The HR was 1.47 (95% CI 0.98–2.20) for overweight women and 2.22 (1.29–3.83) for obese women. The HR was 1.35 (0.96–1.90) for overweight and 1.98 (1.22–3.22) for obesity among both sexes combined. Waist circumference and WHR were not associated with CTS.

In the multivariable analysis, lower clerical workers, entrepreneurs, farmers and manual workers were at a higher risk of CTS than upper clerical workers among both genders combined, men, and women. After an adjustment for confounding factors, regular smoking was associated with CTS among women and both genders combined. We found no statistically significant association between diabetes, rheumatoid arthritis or thyroid diseases and CTS.

There were no interactions between BMI and gender, between BMI and smoking, between BMI and socio-economic status, between BMI and WHR, or between BMI and waist circumference in terms of risk of CTS.

## 4. Discussion

Our study showed that excess body mass is an independent risk factor for CTS. However, this association was statistically significant among women and both genders combined. This finding is in line with those of previously published studies. In 2015, Shiri et al. published a meta-analysis of 58 studies, which revealed that excess body mass increased the risk of CTS and that overweight and obesity were associated with CTS in a dose–response relationship [19].

The mechanisms by which excess body mass increases the risk of CTS are not fully understood. Adipose tissue in the carpal tunnel may tighten the tunnel, leading to median nerve compression [28]. Increased pressure in the carpal tunnel may also decrease blood circulation, leading to median nerve ischemia, demyelination and axonal loss [29]. Another possible mechanism is metabolic syndrome causing median nerve injury by adipose deposition, affecting extracellular protein glycation, mitochondrial dysfunction and oxidative stress [30]. Tenosynovitis in carpal tunnel, caused by inflammation through metabolic syndrome, is also a potential mechanism [31].

As mentioned earlier, a few previously published studies have found waist circumference as a marker of central obesity to increase the risk of CTS in [12,20,21,22]. In the current study, increased waist circumference and WHR were associated with an increased risk of CTS in univariable analysis. However, when we controlled for confounding factors, the associations did not remain statistically significant. As the multivariable analysis of the current study shows, BMI is more relevant than waist circumference and WHR for studying the effect of excess body mass on CTS. It is possible, and even probable, that there is multicollinearity between BMI and waist circumference or WHR. However, including all these variables in the same model (Table 3), it seems that BMI is the strongest of these three to estimate the increased risk of CTS in obesity.

As regards to risk factors for CTS other than those that are weight related, the current study showed that regular smokers, lower clerical workers, entrepreneurs, farmers and manual workers are at a higher risk of CTS than non-smokers or upper clerical workers.

Although previous studies have identified potential risk factors for the development of CTS, the majority of these studies have been cross-sectional. The longitudinal nature of the study better defines the causal relationship. In the current study, the follow-up period was long and the sample size was large. The study population was a representative sample of a single-aged cohort with various socio-economic backgrounds and covered nearly all people born in Northern Finland in 1966. The participation rates in the follow-up studies were also very high. Moreover, the specialized care data on diagnosed CTS that we utilized are reliable and comprehensive, identifying over 95% of hospital discharges and 80–99% of common diagnoses [27].

The current study has some limitations. We used the Care Register for Health Care data from only specialist care. In Finland, the healthcare system is divided into health centers (primary care) and hospitals (specialist care). CTS and suspicion of it are usually coded under the same diagnosis code in primary care. Because of this, we used only hospital data. Thus, using only specialist care data might have excluded patients with mild symptoms and those not willing to visit a hospital. Another limitation of the current study is that the baseline characteristics might have changed over the long follow-up period. Finally, residual confounding may have occurred, and the study did not measure all the risk factors of CTS.

This study showed that BMI is an independent risk factor for CTS and is more relevant than waist circumference or WHR for estimating the effect of excess body mass on the risk of CTS. Future epidemiological studies should investigate whether weight loss as a primary prevention measure decreases the burden of CTS.

## Figures and Tables

**Table 1:** Age-adjusted hazard ratios (HR) with 95% confidence intervals (Cl) of diagnosed carpal tunnel syndrome among men, women and both genders combined (*n* = 9249). NA = not applicable.

Characteristic	Women				Men				Both Genders			
	*n*	Cases	HR	95% CI	*n*	Cases	HR	95% CI	*n*	Cases	HR	95% CI
Sex												
Men					4408	96			4408	96		
Women	4841	194							4841	194	1.95	1.47–2.57
Occupational class												
Upper clerical workers	866	16	1		918	3	1		1784	19	1	
Lower clerical workers	1729	66	1.77	1.00–3.12	780	12	4.49	1.27–15.9	2509	78	2.23	1.32–3.76
Entrepreneurs	265	10	2.74	1.20–6.22	397	8	7.22	1.92–27.2	662	18	3.50	1.80–6.79
Farmers, manual workers	1743	92	3.54	2.02–6.20	2139	71	10.09	3.16–32.2	3882	163	4.53	2.76–7.43
students, pensioners, unemployed	238	10	280	1.25–6.27	174	2	3.82	0.64–22.86	412	12	3.03	1.45–6.32
Body mass index												
Normal	2672	84	1		1798	30	1		4470	114	1	
Overweight	1388	61	1.71	1.00–3.12	1965	45	1.53	0.93–2.52	3353	106	1.64	1.24–2.18
Obese	781	49	2.75	1.86–4.06	645	21	2.52	1.32–4.81	1426	70	2.69	1.92–3.75
Waist circumference according to WHO												
Low risk	2213	73	1		2532	46	1		4745	119	1	
Intermediate risk	1052	43	1.57	1.06–2.31	1010	26	1.73	1.05–2.84	2062	69	1.63	1.20–2.21
High risk	1576	78	2.22	1.55–3.17	866	24	2.16	1.20–3.87	2442	102	2.21	1.63–3.00
Waist-hip ratio according to WHO												
Low risk	1417	53	1		1155	23	1		2572	76	1	
Intermediate risk	1210	51	1.56	1.06–2.29	2286	52	1.45	0.89–2.36	3496	103	1.51	1.11–2.04
High risk	2214	90	1.87	1.28–2.75	967	21	1.88	0.96–3.66	3181	111	1.86	1.34–2.60
Regular smoking												
No	2508	73	1		1896	30	1		4404	103	1	
Yes	2333	121	1.90	1.40–2.59	2512	66	1.73	1.07–2.81	4845	187	1.85	1.42–2.40
Diabetes												
No	4731	187	1		4326	93	1		9057	280	1	
Yes	110	7	1.89	0.89–4.00	82	3	2.28	0.70–7.36	192	10	2.01	1.06–3.78
Thyroid disease												
No	4584	186	1		4346	95	1		8930	281	1	
Yes	257	8	0.93	0.46–1.88	62	1	0.86	0.12–6.22	319	9	0.92	0.47–1.77
Rheumatoid arthritis												
No	4767	191	1		4379	95	1		9146	286	1	
Yes	74	3	1.05	0.34–3.27	29	1	1.72	0.24–12.3	103	4	1.16	0.43–3.12

**Table 2:** Sex-specific and combined sexes’ full model hazard ratios (HR) with 95% confidence intervals (CI) of diagnosed carpal tunnel syndrome for body mass index (BMI), waist circumference and waist-hip ratio (WHR).

Characteristic	Women		Men		Both Genders	
	HR	95% Cl	HR	95% Cl	HR	95% Cl
Model 1. Waist-hip ratio according to WHO						
Low risk	1		1		1	
Intermediate risk	1.48	1.00–2.17	1.30	0.80–2.13	1.39	1.03–1.88
High risk	1.68	1.15–2.47	1.58	0.81–3.09	1.60	1.14–2.24
Occupational class						
Upper clerical workers	1		1		1	
Lower clerical workers	1.57	0.89–2.80	4.31	1.21–15.30	2.02	1.19–3.42
Entrepreneurs	2.36	1.04–5.34	6.76	1.79–25.52	3.12	1.61–6.06
Farmers, manual workers	2.94	1.66–5.20	9.09	2.85–28.96	3.84	2.33–6.34
Students, pensioners, unemployed	2.38	1.05–5.38	3.46	0.59–20.40	2.60	1.24–5.46
Regular smoking						
No	1		1		1	
Yes	1.65	1.21–2.26	1.36	0.84–2.22	1.56	1.19–2.03
Diabetes						
No	NA		NA		1	
Yes					1.75	0.92–3.32
Model 2. Waist circumference according to						
WHO						
Low risk	1		1		1	
Intermediate risk	1.52	1.03–2.24	1.66	1.01–2.73	1.57	1.15–2.13
High risk	2.04	1.42–2.92	1.97	1.10–3.53	1.98	1.46–2.70
Occupational class						
Upper clerical workers	1		1		1	
Lower clerical workers	1.55	0.87–2.76	4.40	1.24–15.61	2.00	1.18–3.39
Entrepreneurs	2.33	1.03–5.28	6.77	1.79–25.56	3.10	1.60–6.02
Farmers, manual workers	2.91	1.65–5.13	9.09	2.85–29.06	3.81	2.32–6.28
Students, pensioners, unemployed	2.32	1.03–5.24	3.39	0.58–19.96	2.55	1.22–5.34
Regular smoking						
No	1		1		1	
Yes	1.65	1.21–2.26	1.37	0.84–2.21	1.56	1.20–2.03
Diabetes						
No	NA		NA		1	
Yes					1.57	0.82–3.01
Model 3. Body mass index						
Normal	1		1		1	
Overweight	1.65	1.17–2.33	1.48	0.90–2.45	1.57	1.18–2.09
Obese	2.48	1.68–3.67	2.21	1.16–4.21	2.35	1.67–3.29
Occupational class						
Upper clerical workers	1		1		1	
Lower clerical workers	1.55	0.87–2.73	4.22	1.19–14.99	1.99	1.17–3.36
Entrepreneurs	2.44	1.08–5.52	6.61	1.75–25.01	3.16	1.63–6.12
Farmers, manual workers	2.85	1.62–5.02	8.90	2.78–28.50	3.75	2.73–6.17
Students, pensioners, unemployed	2.29	1.01–5.17	3.40	0.57–20.19	2.53	1.21–5.31
Regular smoking						
No	1		1		1	
Yes	1.66	1.21–2.26	1.38	0.85–2.24	1.57	1.20–2.04
Diabetes						
No	NA		NA		1	
Yes					1.46	0.76–2.80

**Table 3:** Sex-specific hazard ratios (HR) with 95% confidence intervals (CI) of diagnosed carpal tunnel syndrome. Full model models for men, women and both genders.

Characteristic	Women		Men		Both Genders	
	HR	95% Cl	HR	95% Cl	HR	95% Cl
Occupational class						
Upper clerical workers	1		1		1	
Lower clerical workers	1.53	0.86–2.73	4.31	1.21–15.35	1.98	1.17–3.37
Entrepreneurs	2.44	1.08–5.52	6.69	1.77–25.29	3.16	1.63–6.13
Farmers, manual workers	2.82	1.59–4.98	9.01	2.81–28.84	3.74	2.26–6.17
Students, pensioners, unemployed	2.27	1.00–5.14	3.42	0.58–20.32	2.53	1.20–5.32
Regular smoking						
No	1		1		1	
Yes	1.66	1.21–2.27	1.38	0.85–2.24	1.57	1.20–2.04
Body mass index						
Normal	1		1		1	
Overweight	1.47	0.98–2.20	1.26	0.67–2.39	1.35	0.96–1.90
Obese	2.22	1.29–3.83	1.70	0.63–4.55	1.98	1.22–3.22
Waist circumference according to						
WHO						
Low risk	1		1		1	
Intermediate risk	1.22	0.73–2.03	1.51	0.78–2.92	1.35	0.91–1.99
High risk	1.22	0.62–2.41	1.59	0.59–4.33	1.37	0.78–2.40
Waist-hip ratio according to WHO						
Low risk	1		1		1	
Intermediate risk	1.17	0.76–1.81	0.92	0.54–1.56	1.05	0.75–1.48
High risk	0.97	0.53–1.77	0.74	0.34–1.59	0.87	0.54–1.40
Diabetes						
No					1	
Yes					1.50	0.78–2.88

## Data Availability

NFBC data is available from the University of Oulu, Infrastructure for Population Studies. Permission to use the data can be applied for research purposes via the electronic material request portal. In the use of data, we follow the EU general data protection regulation (679/2016) and Finnish Data Protection Act. The use of personal data is based on cohort participants’ written informed consent at his/her latest follow-up study, which may cause limitations to its use. Please, contact the NFBC project center (NFBCprojectcenter(at)oulu.fi) and visit the cohort website for more information.
